# Psychological and cognitive-emotional moderators of suicidal ideation and self-harm in young adults

**DOI:** 10.1038/s41598-026-37127-4

**Published:** 2026-01-29

**Authors:** Justine Dickhoff, Wenrui Deng, André Aleman, Vera de Vries, Esther Marije Opmeer, Marie-José van Tol

**Affiliations:** 1 Center for Clinical Neuroscience and Cognition, University Medical Center Groningen (UMCG), University of Groningen, Groningen, the Netherlands; 2 Norwegian Artificial Intelligence Research Consortium (NORA), Faculty of Mathematics and Natural Sciences, University of Oslo, Oslo, Norway; 3 Faculty of Psychology and Neuroscience, Maastricht University, Maastricht, the Netherlands; 4 Department of Clinical and Developmental Neuropsychology, University of Groningen, Groningen, the Netherlands; 5 Department of Health and Welfare, Windesheim University of Applied Sciences, Zwolle, the Netherlands

**Keywords:** Suicidal ideation, Self-harm, Entrapment, Mindfulness, Implicit associations, Self-compassion, Diseases, Health care, Psychology, Psychology, Risk factors

## Abstract

The risk for suicide has been conceptualized as a continuum from dysfunctional psychological and cognitive states to suicidal thoughts and behaviour. Little is known about risk and protective factors along the continuum. In the current study, we assessed ninety-four university students (36 individuals indicated suicidal ideation and 33 reported self-harm) for suicidality, depression, entrapment, defeat, hopelessness, mindfulness, self-compassion, and implicit associations with death/suicide. We used regression models and moderation analyses to test if mindfulness, self-compassion, and implicit association with death/suicide related to suicidal ideation, self-harm, entrapment, or moderated the hypothesized continuum from entrapment to suicidal ideation and from entrapment to self-harm. Analyses were corrected for the severity of depressive symptomatology. Results indicated that lower self-compassion and weaker implicit associations with death/suicide were statistically associated with self-harm, but not suicidal ideation. Mindfulness is trend-wise associated with entrapment and moderated the relation between entrapment and suicidal ideation. Self-compassion nor implicit associations with death/suicide moderated the relation between entrapment and suicidal ideation. Hence, lower self-compassion and weaker implicit associations with death/suicide seem directly related to suicide risk behaviour, whereas mindfulness appears to negatively (i.e., suggestive of a protective role) relate to psychological risk-states earlier in the continuum. Therefore, self-compassion and mindfulness seem promising prevention targets for suicide risk in young adults.

**Supplementary Information:**

The online version contains supplementary material available at 10.1038/s41598-026-37127-4.

## Introduction

Suicide is the third leading cause of death between people aged between 15 and 29-year old^1,2^, which is an age range including young people in secondary school and university. The high rates of suicidal thoughts and behaviour in university students across several countries were consistent reported in recent meta-analysis studies^3,4^. In a large-scale epidemiological research of university students, 32.7% indicated that they had thought about suicide at least once in their life, 17.5% indicated lifetime plans, and 4.3% reported at least one suicide attempt in their life^5^. Furthermore, research studies have shown that approximately 15–27% of university students indicated that they had at least once in their lives intentionally harmed their own body either with or without the intention to die from it^6–8^. Of note, individuals engaging in self-harm were at higher risk of subsequent suicide behavior^9,10^. The high numbers of students with suicidal thoughts and/or self-harm create an imperative need to study factors that can protect young adults in the undergraduate phase from developing suicidal tendencies.

So far, predicting future risk for suicide and offering targeted treatment has been a clinical challenge, most likely reflecting that suicide results from a complex interplay of various risk factors, including factors that increase the likelihood of an individual dying by suicide, and protective factors that offer potential alleviation^11,12^. Previous theoretical models have suggested that some factors are more closely linked to a suicide attempt whereas others have a more indirect association with suicide attempts. In the Integrated Motivational-Volitional (IMV) Model of Suicidal Behaviour by O’Connor^11^ and O’Connor & Kirtley^13^, suicidal behaviour has been hypothesized to progress through three successive phases starting with the *premotivational phase* (largely determined by the biopsychosocial context), followed by the *motivational phase*, and finally the *volitional phase.* The *motivational phase* is proposed to start with the feeling of being defeated which can subsequently lead to induced feelings of entrapment, and feelings of entrapment can transition to the emergence of suicidal ideation. In the final phase of the IMV-Model – the *volitional phase*, individuals that transition from suicidal ideation to suicidal behaviour, would be placed. The progress from one phase to the next in the IMV-Model is influenced by various moderators, which can either expedite or buffer the transition.

Moderators may include the propensity to better recognize, attribute, and regulate emotions, abilities that have been linked to lower risk of suicidal thoughts and/or behaviours^14–16^. Consequently, by bolstering these processes, the risk of suicide potentially reduces^17–19^, thereby protecting against suicidal tendencies. Two effective approaches that enhance emotion regulation strategies are the practice of mindfulness and the cultivation of self-compassion^20–23^. Mindfulness can be characterized by non-judgmental awareness of an individual’s moment-to-moment emotions and thoughts^24^. Self-compassion, a concept closely associated with mindfulness, encompasses a sense of understanding and kindness toward oneself, particularly during instances of failure^25^. These two approaches when practiced, teach individuals to be non-judgemental and in control of their own emotions and thoughts^24,26^. Both self-compassion and mindfulness have been found to reduce depression and suicidal ideation among adolescents and adults with suicidal risk independently^27,28^. However, the relations of the two putative protective skills have not been studied in the context of the IMV, especially at the earlier stage of the continuum (e.g., entrapment). Elucidation on these relations might provide a clearer understanding of which protective factors influence which stages of the suicidal continuum.

In the past two decades, the practice of mindfulness has gained significant attention as a technique to improve mental health. In several studies involving post-secondary students, the positive effect of mindfulness on alleviating symptoms of depression, anxiety, and stress has been demonstrated^29,30^. Furthermore, mindfulness-based interventions, as well as naturalistically observed mindfulness thought patterns (independent of a received treatment) have been associated with lower suicidal thoughts in undergraduate students^31,32^ and have been shown to positively affect adolescents and adults with self-harm behaviours^33–35^. However, knowledge about the relation between risk factors recognized in the IMV-Model (i.e., entrapment, ideation, self-harm) and mindfulness is limited. Studies with young adults experiencing suicidal ideation suggested that mindfulness-based therapy decreased the feelings of defeat and entrapment, and suicidal ideation^36,37^. Entrapment is closely related to the development of suicidal behaviour^13^, therefore mindfulness might possibly also act as a buffer in the transition from risk factors of suicide to development of suicidal ideation and/or self-harm. This is supported by a study with undergraduate students with depressive symptoms, where individuals with thoughts of burdensomeness, indicated lower levels of suicidal thoughts if they had higher mindfulness levels^38^. To the best of our knowledge, no study so far, has examined whether mindfulness also acts as a moderator in the association between entrapment and suicidal ideation or self-harm.

Similar to mindfulness, self-compassion has shown positive effects on improving depressive symptoms and well-being^39,40^, but also has moderate relations with various risk factors for suicide, such as suicidal ideation, self-harm^41,42^, depressive symptoms^42,43^, and daily hassles^44^. However, limited studies have been conducted to investigate the risk factors of the IMV-Model and self-compassion. Self-compassion was found to negatively relate to feelings of entrapment in university students^45^. Additionally, self-compassion total scores were found to mediate the relation between defeat and entrapment and its self-judgement and isolation subscale scores to mediate the relationship between entrapment and suicidal ideation (part of the motivational phase of the IMV-Model) in an online survey study of a large community sample (*n* = 698)^46^. No study so far investigated both mindfulness and self-compassion in relation with risk factors of the IMV-Model. Mindfulness and self-compassion have different elements, with mindfulness emphasizing metacognition, attention regulation, and awareness, self-compassion focuses on self-care and acknowledging that painful experiences are part of being human^25^. Self-compassion and mindfulness may therefore have distinct associations with suicidal risk factors.

Furthermore, a comprehensive understanding of emotional distress associated with suicidal behaviour would benefit from the identification and examination of unconscious emotions and beliefs about ourselves and others outside of our conscious control. To evaluate the underlying emotions associated with suicidal tendencies, we require objective markers that go beyond self-reports or interviews and delve into measures exploring the implicit processes. A potentially suitable candidate for this purpose is the death/suicide implicit association test (d/s-IAT), which measures implicit associations with suicide without an explicit disclose or awareness^47^. The IAT is a computerized reaction time test which measures the strength of automatic associations an individual has with certain topics^47^. Earlier studies have shown that the IAT containing words related to death/suicide was able to reveal reported suicidal ideation in a sample of undergraduate students^48^ as well as in adolescents with a psychiatric disorder^49^. Similar results were observed for self-harm behavior in a large adults community sample^50^, in a sample with undergraduate students^48^, as well as in a samples with adolescents^51^ and young adults with psychiatric disorders^52,53^, though not consistently^49,54,55^. Furthermore, in an adult inpatient population, suicidal ideation, hopelessness, and depression severity were found to link to higher implicit associations with death/suicide^56^. Inconsistent results have also been observed, no relation was found between implicit associations with death/suicide and suicidal thoughts or behaviour among depressed adult inpatients^57^. Given the inconsistency in research findings, there is a need to further investigate the relation between implicit associations and the suicidal continuum as described in the IMV-Model. We aimed to investigate the potential hypothesis, as posed by O’Connor & Kirtley^13^, that higher levels of automatic processes related to suicidal desires may expedite the transition from entrapment to suicidal ideation. Furthermore, we explored the role of mindfulness, and self-compassion in this relation.

Given the need for effective prevention of suicidal thoughts and behaviour in university students, we aimed to investigate the relation between the suicidal risk factors, suicidal ideation and self-harm (which includes individuals with and/or without the intention to die through self-harming) on the one hand, and psychological risk-moderators mindfulness, self-compassion, and implicit associations with death/suicide on the other hand. First, we investigated the direct associations between the proposed risk and protective factors and suicidal ideation and self-harm. We hypothesize that lower levels of mindfulness and self-compassion and stronger implicit associations with death/suicide would be associated with suicidal ideation and self-harm. Second, we examined if the proposed factors were linked to entrapment, a suicide risk factor associated with earlier stages of the IMV-Model. We hypothesize that lower mindfulness and self-compassion, and stronger implicit associations with death/suicide would be associated with feelings of entrapment. Finally, we examined whether the proposed risk and protective factors acted as psychological moderators in the connection between feelings of entrapment and suicidal ideation. We hypothesize that mindfulness, self-compassion, and implicit associations with death/suicide would serve as moderators in this relationship.

## Methods

### Participants and procedure

For this cross-sectional study, participants were recruited via a research-participant platform for first-year psychology students at the University of Groningen from 2019.3 to 2019.6. The study was approved by the ethical committee of the University of Groningen (No.18288-S) and was performed in accordance with the Declaration of Helsinki. Before entering the study, each participant received written and oral information about the study from a researcher trained in suicide research. After agreeing with the terms of the study, the participants and researcher signed the informed consent and started with the psychological tasks and questionnaires. During participation the researcher stayed in the same room to answer possible questions. Participants were rewarded with study credits after completing the research. Owing to the sensitive topic of the study, in addition participants received contact information from the Dutch Suicide Prevention Service (113 online) and the researcher asked every participant how they were feeling after completing the study.

### Measures

#### The suicide probability Scale – Suicidal ideation subscale (SPS-SIS)

We used the *‘Suicidal Ideation Subscale’* of the Suicide Probability Scale^58^ to assess severity of current suicidal ideation. The subscale consists of eight items that measure thoughts and behaviours related to suicide (e.g., “I think of suicide”). On an ordinal scale, participants had to indicate how often the item applies to them (ranging from 0 = “Non or little of the time” to 3 = “Most or all of the time”). The subscale has shown good validity^59^. We used a sum score for the current study. The SPS-SIS showed good internal consistency in the current sample (Cronbach’s α = 0.82).

#### The VOZZ screen

The Questionnaire assessing Suicide and Self Injury (in Dutch: *Vragenlijst over Zelfdoding en Zelfbeschadiging* (VOZZ)) has been designed as a signaling tool for suicidality among youth (aged 12–21 years) with high internal consistency (Cronbach’s α = 0.89)^60^. We used the screening variant of the VOZZ, which consists of ten items assessing thoughts and actions about self-harm, suicide and life-events. For the current study, we used three items of the VOZZ separately to indicate suicidal ideation (translated: I thought about ending my life), self-harm (translated: I have deliberately injured, cut or scratched myself at one or more occasions) and suicide attempt (translated: I have attempted suicide). Answers on the items indicate the frequency respondents experience these in life (ranging from 0 = “never” to 4 = “very often). This short-form scale demonstrated a Cronbach’s alpha of 0.68 in the current sample, suggesting marginally acceptable internal consistency.

#### The entrapment scale

The Entrapment Scale^61^ has been used frequently to study the concept of entrapment and requires individual to rate internal and external feelings of entrapment on 16 items on a five-point Likert scale (ranging from 0 = “not at all like me” to 4 = “extremely like me”). Gilbert & Allan (1998)^61^ reported high levels of internal consistency in the student population, with Cronbach’s alphas of 0.93 on the subscale for internal entrapment and 0.88 for the subscale of external entrapment. For the current study, the total scores of the Entrapment Scale (internal and external subscales combined) demonstrated excellent internal consistency in the current sample (Cronbach’s α = 0.94).

#### The five facet mindfulness questionnaire (FFMQ)

The FFMQ^62^ is a self-report scale with 39 items that measures five key mindfulness skills (non-reactivity to inner experience, observing/noticing, acting with awareness, describing and non-judging of experience) on a 5-point Likert scale (1 = never or very rarely true to 5 = very often or always true). In the current study, we calculated a total score of all items to reflect a global mindfulness score. The FFMQ has been shown to be a reliable and valid scale to measure mindfulness in a student population^62,63^. The FFMO showed high internal consistency in the current sample with a Cronbach’s alpha of 0.90.

#### Self-compassion scale

The Self-Compassion Scale^64^ measures emotions, thoughts, and behaviours related to self-compassion. The Dutch version consists of 24 items and assesses self-compassion on a 5-point Likert scale from 1 = “almost never” to 5 = “almost always”^65^. In the present study, the total score across all items for each participant was calculated, divided by number of items and used to measure their overall level of self-compassion^25^. The Self-Compassion Scale demonstrated excellent internal consistency in the current sample (Cronbach’s α = 0.94).

#### Death/suicide implicit association test (d/s IAT)

The d/s IAT^47^ is a word sorting task for which individuals have to indicate as fast as possible the membership of certain words related to oneself or others and words referring to death or life. The two categories (1. oneself/others and 2. life/death) appeared in either the left or right corner of the computer screen and a sequence of words related to these categories appeared in the middle of the screen. The IAT measures whether one has more automatic associations with either death or life based on response latencies (see the supplement for the stimuli and block sequence). The relative strength of each participant’s association between “death” and “me” was indexed by calculating a D-score (the standardized score used for the calculation of within-person differences) for each participant^66^. A higher D-score indicates stronger implicit associations between the self and death/suicide and therefore a higher level of implicit suicidal ideation. A score of zero indicates no implicit associations. IATs have been found to have good reliability^67^ and construct validity^68^. The d/s IAT shows good reliability^50,69^ and mixed results for validity^48,50,56,69,70^.

#### Classification of suicidal ideation and self-harm

We stratified participants based on whether they had suicidal ideation or engaged in self-harm behaviors. A score higher than zero on the SPS-SIS total score and additionally the item measuring suicidal ideation of the VOZZ was used to indicate individuals with *suicidal ideation* in the sample of the current study. We used a score of zero on both the SPS-SIS and the item of the VOZZ regarding suicidal ideation to indicate individuals with *no suicidal ideation*. The total score of the SPS was additionally used to indicate severity of suicidal ideation. Individuals with self-harm were classified with the item measuring self-harm from the VOZZ. A score higher than zero on this item was used to define individuals with *self-harm*, and a score of zero classified individuals with *no self-harm*.

#### Additional measures

To provide a descriptive context of clinical and cognitive constructs with relevance for suicidal behavior, we additionally assessed the Beck Hopelessness Scale^71^, the Defeat Scale^61^, the Inventory of Depressive Symptomatology (IDS)^72^. The Reading the Mind in the Eyes Task (RMET) was also administered during the assessment session to establish normative response patterns for social-emotional recognition in the current sample^73^. Given that the task does not have objectively correct answers, it was included to determine the most commonly selected responses, which we plan to use as reference values for future studies. The RMET was not included to address our primary research question. Additional information on these measures is provided in the supplement.

### Statistical analysis

SPSS Statistics 23 was used for the analysis. T-tests and Chi-Square tests were used to check for group differences in age, sex, and study constructs. *Pearson correlations* (bivariate) were calculated to explore the strength of simple correlations between all constructs included in the study.

Because depression severity has been related to mindfulness, self-compassion, d/s IAT, suicidal ideation, and self-harm^74,75^, we included the IDS-total score (minus suicidality item) as covariates in all following analyses. Multicollinearity among mindfulness, self-compassion, d/s IAT, and depression was checked using Variance Inflation Factors (VIFs; values above 10 indicate serious multicollinearity)^76^. All variables showed acceptable VIF (1.09–2.45), indicating no significant multicollinearity.

#### Hypothesis 1

Lower Mindfulness, self-compassion and stronger implicit associations with death/suicide are associated with suicidal ideation and self-harm.

*Two binary logistic regression analyses* were performed with (1) suicidal ideation (yes/no) and (2) self-harm (yes/no) as the dependent variable and the following predictors: mindfulness, self-compassion, and the d/s IAT. For the logistic regression models the following assumptions were tested: linear relationship between the dependent variable (the logit) and independent variables, normal distribution of the residuals, outlier detection.

#### Hypothesis 2

Higher mindfulness, self-compassion, and weaker implicit self-associations with death/suicide are associated with lower feelings of entrapment.

A *linear regression model* was set up with entrapment as dependent variable and mindfulness, self-compassion, and the d/s IAT as predictor variables. For the linear regression models, R-Square tests were used to test the percentage of variance that can be explained by the predictor variable/s. For the linear regression models the following assumptions were tested: linear relationship between dependent variable and independent variables, normal distribution of the residuals, outlier detection.

#### Hypothesis 3

Mindfulness, self-compassion, and implicit associations with death/suicide are statistical ‘motivational’ moderators of the relation between entrapment and suicidal ideation.

Using *moderation analyses* we explored if mindfulness, self-compassion, and the d/s IAT moderated the relation between entrapment (independent variable) and suicidal ideation (dependent variable) to understand which individuals are at higher suicidal risk. Entrapment is an independent variable here (compared to Hypothesis 2), because we tested the effect it has on suicidal ideation, together with the moderators. We conducted three moderator analyses for which we used PROCESS macro version 3.4. for SPSS ^77^. In PROCESS moderation effects are tested with the use of regression models with interaction effects (formula: Y′= b0 + b1 × 1 + b2 × 2 + b3(X1×X2)+ε), the interaction effects indicate how the moderating variable affects the relation between the dependent and independent variable. The moderation analyses (PROCESS macro model 1; simple moderation) automatically detect and transform categorical outcome variables (suicidal ideation vs. no suicidal ideation in our study) into dummy variables, and then consequently use logistic regression models (z-statistics). Bootstrapping, of 10.000 resamples (as recommended by Hayes, 2013^77^) was used to create confidence intervals.

Multiple comparison correction was applied to correct the alpha-level for the number of tests performed, while taking the interdependency between dependent variables into account (Quantitativeskills. Hilversum (NL): Internal Publication, 2019; https://www.quantitativeskills.com/sisa/). Resulting alpha levels were *α* = 0.025 for the Pearson Correlation analysis between all constructs of the study (mean *r* = .35, corrected for two tests to adjust for the two variables of interest: suicidal ideation and self-harm) and *α =* 0.036 for the logistic regression models (Hypothesis 1; suicidal ideation and self-harm show a Spearman’s *r* = .532). An *α = 0.05* was used for the entrapment analyses (Hypotheses 2) and *α* = 0.0167 for the moderator analyses (Hypotheses 3; Bonferroni corrected for three tests).

## Results

### General results

Ninety-four participants (63 women, 31 men; Age: M: 19.91, SD ± 1.6, see Table 1) completed the study. All participants completed pre-university diplomas or equivalent qualifications.

Thirty-six indicated suicidal ideation (SI; i.e., the SPS-SIS total score and the VOZZ suicidal ideation item score were both higher than zero) and 34 individuals indicated no suicidal ideation (noSI; i.e., both the SPS-SIS total score and the VOZZ suicidal ideation item score were zero). Twenty-four individuals showed inconsequent SI (not meet the dual criteria of SI) and were therefore not included in the analysis investigating suicidal ideation. Of the 36 participants in the SI group, 21 also had a history of self-harm and six had a history of suicide attempts (six reported both self-harm and attempts).

Thirty-three individuals reported self-harm (SH; i.e., participants who scored the VOZZ self-harm item higher than zero) and of these, 21 reported SI. 59 individuals indicated never engaging in self-harm (noSH; i.e., VOZZ-SH item was scored zero). Two individuals did not report on the self-harm item and therefore not included in the self-harm analysis. Six individuals reported a suicide attempt lifetime, all six also reported SI and self-harm.

Individuals with SI vs. noSI and self-harm vs. no self-harm did not differ in age and sex. Not all individuals completed all measurements, therefore sample sizes varied slightly for some of the scales and/or tests. Owing to technical difficulties data was missing for some participants (d/s IAT: *n* = 2, IDS: *n* = 1, defeat-scale: *n* = 3). No imputation was conducted because missing values were low in number.

**Table 1:** Sample characteristics and group differences on the study constructs.

	Suicidal Ideation (*n* = 36)		No Suicidal Ideation (*n* = 34)			Self-Harm (*n* = 33)		No Self-Harm (*n* = 59)		
	Mean ± SD	95% - CI	Mean ± SD	95% - CI	Stats	Mean ± SD	95% - CI	Mean ± SD	95% - CI	Stats
Demographics										
Age	20.22 ± 1.6	19.7–20.8	19.76 ± 1.7	19.2–20.3	n.s.	20.21 ± 1.6	19.7–20.8	19.71 ± 1.6	19.3–20.1	n.s.
Sex, female, N (%)	27 (79.4%)	-	21 (58.3%)	-	n.s.	39 (66.1%)	-	23 (69.7%)	-	n.s.
Study Constructs										
Severity of Suicidal Ideation^1^	3.9 ± 2.8	3.0–4.9	0.0 ± 0.0	0.0–0.0	**	3.0 ± 3.0	2.0–4.1	1.2 ± 2.0	0.6–1.7	**
Severity of Self-Harm^2^	2.1 ± 1.2	1.7–2.6	1.1 ± 0.4	0.97–1.27	**	2.7 ± 0.8	2.4–3.0	1.0 ± 0.0	1.0–1.0	**
Defeat	23.0 ± 11.0	19.4–27.6	7.2 ± 5.7	5.2–9.2	**	21.0 ± 12.2	16.8–26.4	12.0 ± 9.6	9.3–14.7	**
Entrapment	21.2 ± 13.2	18.0–27.3	7.2 ± 5.7	1.2–5.8	**	17.9 ± 13.7	13.6–24.2	8.4 ± 10.7	5.8–11.9	**
Hopelessness	6.0 ± 3.6	5.0–7.6	2.3 ± 2.1	1.5–2.9	**	5.6 ± 4.0	4.2–7.4	3.4 ± 2.7	2.7–4.2	*
Depression^3^	21.6 ± 12.7	17.6–26.2	8.5 ± 6.2	6.1–10.0	**	21.2 ± 12.4	18.7–29.3	11.6 ± 8.7	7.9–13.2	**
Mindfulness	118.1 ± 17.5	112.1–124.6	135.5 ± 16.1	131.3–142.5	**	124.6 ± 18.7	118.4–131.9	129.1 ± 18.01	124.7–135.0	n.s.
Self-Compassion	3.5 ± 0.8	3.2–3.8	4.6 ± 1.0	4.2–5.0	**	3.5 ± 0.9	3.2–3.8	4.3 ± 1.0	4.0–4.6	**
d/s IAT ^4^	-0.3 ± 0.4	-0.5 – -0.1	-0.4 ± 0.3	-0.5 – -0.3	n.s.	-0.5 ± 0.3	-0.6 – -0.3	-0.3 ± 0.6	-0.4 – -0.2	*

Note. * indicates *p* < .05. **indicates *p ≤ .001.* n.s. indicates statistically non-significant. ^1^measured with the SPS-SIS total score. ^2^VOZZ self-harm item score. Two individuals did not report on the self-harm item and therefore not included in the self-harm analysis. ^3^ Inventory for Depressive Symptomatology minus item-16 about suicidal ideation. ^4^a negative d/s IAT score reflects a tendency to associate oneself more with life than with death.

### Correlation analyses

Suicidal ideation (yes/no), self-harm (yes/no), defeat, entrapment, hopelessness, and depressive symptoms were all positively correlated (*r*s > 0.23, *p*s < 0.025, see Table 2), and all showed a negative correlation with mindfulness (*r*s>-0.44, *p*s < 0.001; except for self-harm) and self-compassion (*r*s>-0.37, *p*s < 0.001).

The d/s IAT a showed a negative correlation with mindfulness (*r=-.28*, *p = .006*), but not with any other study constructs (*p*s > 0.03).

**Table 2:** Correlation analyses between defeat, entrapment, hopelessness, depressive symptoms, self-compassion, mindfulness, d/s IAT, suicidal ideation and self-harm.

	1	2	3	4	5	6	7	8	9
1. Suicidal Ideation (yes/no)^**1**^	1								
2. Self-Harm (yes/no)^**1**^	0.53**	1							
3. Defeat^**2**^	0.71**	0.36*	1						
4. Entrapment^**2**^	0.71**	0.36**	0.82**	1					
5. Hopelessness	0.55**	0.23*	0.85**	0.76**	1				
6. Depressio^**2 ,3**^	0.60**	0.43**	0.81**	0.77**	0.72**	1			
7. Self-Compassion	− 0.59**	− 0.37**	− 0.72**	− 0.65**	− 0.62**	− 0.64**	1		
8. Mindfulness	− 0.44**	− 0.10	− 0.61**	− 0.59**	− 0.52**	− 0.43**	0.66**	1	
9. d/s IAT	0.12	− 0.23	0.13	0.18	0.11	0.15	− 0.23	− 0.28*	1

Note. * indicates *p* < .025 **indicates *p* < .001. ^1^Spearman rank correlations were used. ^2^Additionally, Spearman rank correlations were conducted since data was not normal distributed, significance did not change. ^3^ Depression was assessed with the Inventory for Depressive Symptomatology (self-report, minus the item-16 about suicidal ideation).

### Logistic and linear regression models

#### Associations between suicidal ideation (yes/no) with mindfulness, self-compassion, d/s IAT and depression

Assumptions were not violated. The logistic regression for suicidal ideation was statistically significant (χ^2^(4) = 34.33, *p* < .001), explained 53.5% (Nagelkerke’s *R*^*2*^) of the variance in SI and classified 79.1% of the cases correctly. Only depressive symptoms added significantly to the model (*p* < .014). Mindfulness, self-compassion, and the d/s IAT did not statistically predict SI in this model (*p >* .06, see Table 3).

#### Associations between self-harm (yes/no) with mindfulness, self-compassion, d/s IAT and depression

The logistic regression for self-harm was statistically significant (*χ*^2^(4) = 33.51, *p* < .001), explained 43.0% (Nagelkerke’s *R*^*2*^) of the variance in self-harm and classified 83.1% of the cases correctly. D/s IAT scores, self-compassion, and depressive symptoms added significantly to the prediction of self-harm (all *ps* < 0.02; see Table 3), the d/s IAT decreased the log odds for self-harm by -3.06 (*p* < .002), self-compassion decreased the log odds for self-harm by 1.01 (*p* < .021), whereas depression increased the log odds for self-harm by 0.09 (*p =* .01).

Mindfulness did not predict self-harm in this model (*p >* .036, see Table 3).

#### Associations between entrapment with mindfulness, self-compassion the d/s IAT and depression

The model including mindfulness, self-compassion, the d/s IAT, and depression (predictors) for predicting entrapment was significant (F_(4,86)_ = 49.02, *p* < .001) and indicated that our model could explain 69.5% (R^2^) of the variance in entrapment total scores. Mindfulness and depressive symptoms added significantly to the model, for every unit increase in mindfulness, entrapment decreased by 0.21 (*p* < .001) and for every unit increase of depression, entrapment increased by 0.70 (*p* < .001) while self-compassion and the d/s IAT were no significant predictors of entrapment (see Table 3).

Existing literature suggests that internal entrapment is a stronger predictor of suicidal ideation than external entrapment, therefore the specific effects of internal and external entrapment should be considered^78^. Therefore, we ran the model separately for internal and external entrapment to test whether mindfulness, self-compassion and the d/s IAT have specific effects on internal/external entrapment. The results showed that both mindfulness and depressive symptoms statistically significantly predicted internal entrapment (R^2^ = 0.594, F_(4,86)_ = 31.41, *p* < .001) as well as external entrapment (R^2^ = 0.719, F_(4,86)_ = 55.04, *p* < .001) (see Table 3).

**Table 3:** Logistic regression and linear regression models between suicidal ideation, self-harm, (internal/external) entrapment and mindfulness, self-compassion, the d/s IAT and depression.

Logistic regression models/ Hypothesis 1							
Predictor	β^1^	S.E.	OR^2^	Wald’ χ2	*p*	95% CI for Odds Ratios	
						Lower	Upper
Suicidal Ideation (yes/no)							
Constant	3.54	3.24	34.39	1.19	0.275		
Mindfulness	-0.05	0.03	0.95	3.50	0.062	0.90	1.00
Self-Compassion	0.27	0.53	1.31	0.26	0.612	0.46	3.71
d/s IAT	0.28	0.87	1.32	0.10	0.750	0.24	7.26
Depression	0.17	0.07	1.19	6.05	0.014 *	1.04	1.36
Self-Harm (yes/no)							
Constant	-2.20	2.41	0.11	0.83	0.362		
Mindfulness	0.02	0.02	1.02	1.43	0.232	0.99	1.07
Self-Compassion	-1.01	0.44	0.36	5.34	0.021*	0.15	0.86
d/s IAT	-3.06	1.01	0.05	9.21	0.002*	0.01	0.34
Depression	0.09	0.04	1.10	6.62	0.010*	1.02	1.18

Linear regression models/ Hypotheses 2							
Predictor	β ^3^	S.E.	Beta^**4**^	t	*p*	95% CI	
						Lower	Upper
Total Entrapment							
Constant	31.45	6.64	-	4.74	< 0.001*	18.26	44.64
Mindfulness	− 0.21	0.06	− 0.30	-3.68	< 0.001*	− 0.32	− 0.10
Self-Compassion	− 0.87	1.14	− 0.07	− 0.76	0.450	-3.13	1.40
d/s IAT	− 0.17	2.10	− 0.01	− 0.08	0.936	-4.35	4.01
Depression	0.70	0.09	0.60	7.82	< 0.001*	0.53	0.88
Internal entrapment							
Constant	17.98	4.54	-	3.96	< 0.001 *	8.95	27.02
Mindfulness	− 0.10	0.04	− 0.25	-2.70	0.008 *	− 0.18	− 0.03
Self-Compassion	− 0.67	0.78	− 0.09	− 0.86	0.391	-2.23	0.88
d/s IAT	0.29	1.44	0.01	0.20	0.842	-2.57	3.15
Depression	0.38	0.06	0.55	6.15	< 0.001 *	0.26	0.50
External entrapment							
Constant	13.47	2.83	-	4.76	< 0.001 *	7.84	19.10
Mindfulness	− 0.10	0.02	− 0.34	-4.29	< 0.001 *	− 0.15	− 0.06
Self-Compassion	− 0.19	0.49	− 0.04	− 0.40	0.693	-1.16	0.78
d/s IAT	− 0.46	0.90	− 0.03	− 0.51	0.612	-2.24	1.33
Depression	0.33	0.04	0.63	8.45	< 0.001 *	0.25	0.40

Note. * indicates *p < .*036 for the logistic regression models (Hypothesis 1) *and p < .*05 for the entrapment analyses (Hypotheses 2).

^1^Standardised beta coefficient. Predicted change in log odds for every unit increase or decrease on the predictor variable.

^2^OR (odds ratios), indicates change in odds for every unit increment in our predictor variable. A variable greater than one indicates an increase in the odds of having SI.

^3^ Standardized beta coefficients.

^4^Unstandardised beta coefficient.

### Moderator models

**Fig. 1:**
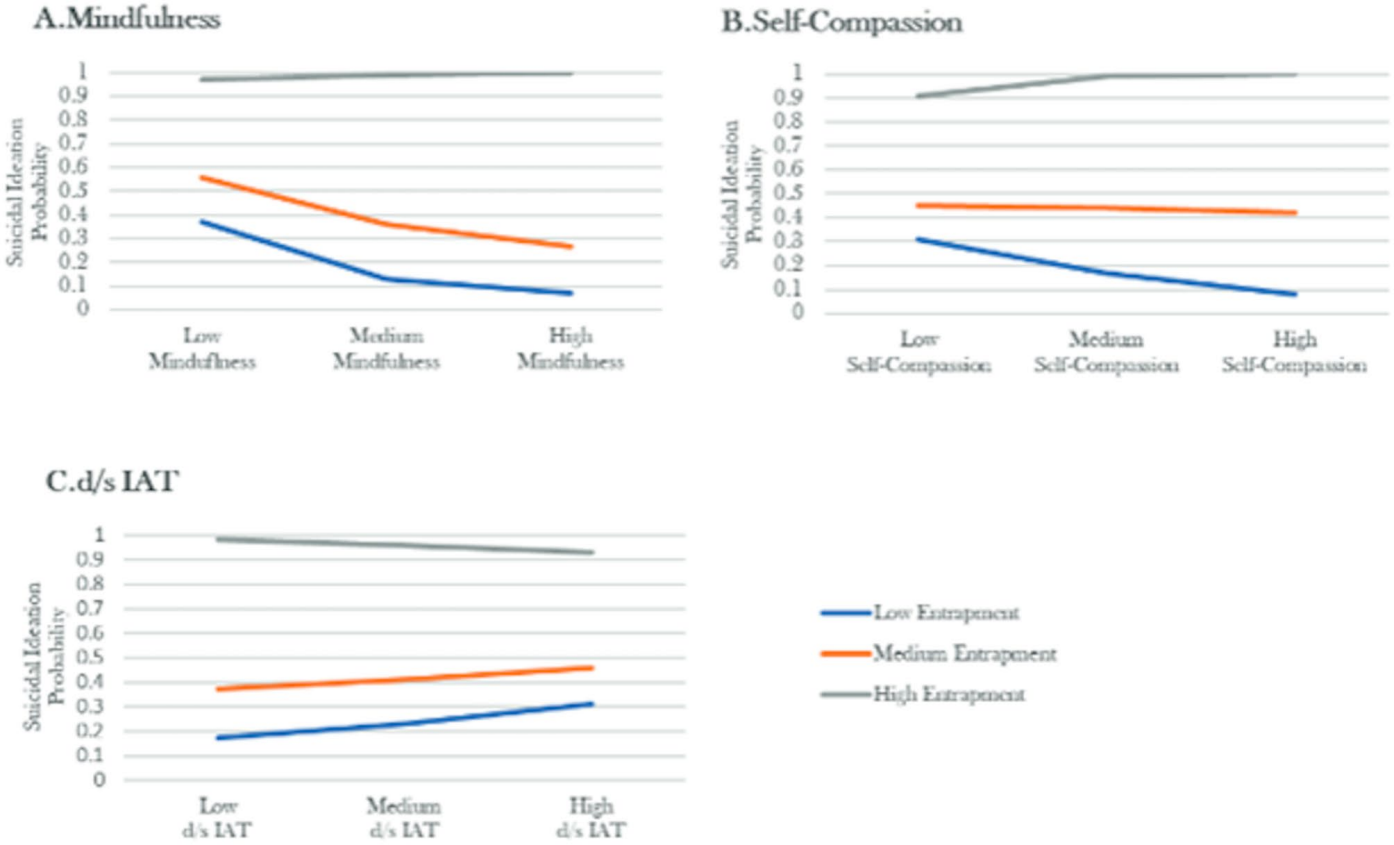
Simple slope plots for the moderation analyses between suicidal ideation and entrapment with (**A**) mindfulness, (**B**) self-compassion and (**C**) d/s IAT as moderators. Note. As recommended by Hayes (2013)^77^ low medium and high scores of the dependent and moderator variables indicate the 16th, 50th, and 84th percentiles. To enhance interpretability, the probability of suicidal ideation is used instead of the log odds ratio. All analyses were adjusted for depression severity.

The moderation effects can be found in Fig. 1. Self-compassion (*p* = .069, b = 0.10, z = 1.81) and the d/s IAT (*p* = .418, b = − 0.08, z = − 0.81) did not moderate the relation between the total entrapment and SI. Mindfulness was found to moderate the relation between the total entrapment and SI, but did not survive correction for multiple comparisons (*p* = .042, b = 0.004, z = 2.03).

As explorative analyses, we ran the moderation models separately on the relation between internal/external entrapment and SI to test whether mindfulness, self-compassion and the d/s IAT have distinct moderation effects, with depression as a covariate (see supplementary Table). In terms of the relation between external entrapment and SI, mindfulness showed significant moderation effects (*p* = .047, b = 0.009, z = 1.99).

#### Post-hoc explorative analyses: moderation analysis modeling the relation between suicidal ideation and self-harm with self-compassion and implicit associations as moderators

Because we observed a relation between self-compassion, d/s IAT, and self-harm, we explored whether self-compassion and d/s IAT are potential volitional moderatorsof the relation between suicidal ideation and self-harm. No effect was found for the d/s IAT (*p* = .15) or self-compassion (*p* = .60) as moderator variables between SI and self-harm (see supplementary Figure).

## Discussion

The aim of the present study was to investigate risk and protective factors for suicidal ideation and self-harm in a population of young adults. First, we found lower levels for self-compassion to relate to self-harm but not suicidal ideation, whereas mindfulness was not associated with suicidal ideation nor self-harm. Interestingly lower implicit associations with death/suicide related to the presence of self-harm, but no relation was found between suicidal ideation and d/s IAT. Second, lower levels of mindfulness was associated with entrapment, but no association was found for self-compassion and implicit associations with death/suicide (d/s IAT). Lastly, moderation models were conducted to test if mindfulness, self-compassion and the d/s IAT moderated the relation between entrapment and suicidal ideation, and if self-compassion and the d/s IAT moderated the relation between suicidal ideation and self-harm. Subthreshold, mindfulness was found to be a weak moderator of the relation between entrapment and SI. Inspection of the moderation suggests that at lower levels of entrapment, mindfulness was associated with lower severity of ideation, whereas at highest levels of entrapment, such ameliorating effects were not observed. However, the moderation effect did not survive correction for multiple comparisons, making this effect in need of replication in larger and/or clinical samples to assess its clinical significance. Self-compassion and the d/s IAT were not identified as moderators between entrapment and SI nor between SI and self-harm.

Both mindfulness and self-compassion were associated with risk factors for suicide. For mindfulness, this was limited to a key risk factor of earlier stages or psychological states in the suicidal process, namely entrapment. Though in simple correlation analyses, mindfulness related to suicidal ideation, in the regression model, mindfulness was not a significant predictor of suicidal ideation. Low self-compassion was associated with self-harm, both in the simple correlation analyses, and in the regression model controlled for depressive symptom severity. Previous studies found mindfulness as well as self-compassion to link to suicidal ideation, self-harm and other risk factors (such as depression severity or thwarted belongingness) for suicide^31,33,37,41,45,79–81^. From the present results, it could be inferred that mindfulness might enable an individual to develop a non-judgmental awareness of persistent negative thoughts, which could aid in disrupting the ruminative cycle, a pattern frequently observed in individuals experiencing suicidal feelings^82,83^. On the other hand, targeting self-compassion techniques, including enhancing self-love and appreciating one’s own body might help in preventing self-harm.

We observed no relation between suicidal ideation or entrapment and the d/s IAT, whereas for self-harm behavior we did identify a relation with lower implicit associations. Contrary to our results, other studies found a link between stronger implicit associations with suicide and self-harm behavior in a large adults community sample^50^, in a sample with undergraduate students^48^ as well as in a samples with adolescents and young adults with psychiatric disorders^52,53^. However, other studies reported no differences in implicit associations with death/suicide to be observed between lifetimes self-harm behavior and no self-harm behavior in adolescents with a psychiatric disorder^54^. The negative relation between implicit associations with death/suicide and self-harm in our study was unexpected, as we expected individuals with self-harm to show stronger implicit associations with death/suicide compared to individuals without self-harm behaviour. Weak implicit associations with death and suicide might indicate that individuals with self-harm behaviour hold conflicting intention of death, therefore they do not show strong implicit identification with death-related materials. The variance in results could potentially be clarified by considering the composition of the studied group, particularly, our assessment on self-harm did not distinguish self-harm behaviours with or without intention to die. These behaviours differ in both function and motivational mechanisms^84^. Non-suicidal self-injury behaviour (NSSI) is frequently used as an emotion regulation strategy to relieve unpleasant emotions, rather than to end life^85,86^. Therefore, individuals who self-harm without suicidal intention may not identify themselves with death/suicide and show a weaker association. The existence of participants with NSSI behaviours in our sample may contribute to the weaker death/suicide association. Future research should distinguish NSSI from self-harm with lethal intention to test their differential relationship with implicit association with death and self-harm stimuli. Furthermore, the comparatively milder and less frequent occurrences of self-harm behaviours in our sample may also influence the current finding.

Our findings on the association between suicidal ideation and the IAT also differ from prior investigations. Some earlier studies, involving undergraduate students^48^ and adolescents with psychiatric conditions^49^ established a connection between suicidal ideation and implicit associations with suicide or death. It is plausible that the degree of suicidal ideation in our studied population might not have been robust enough to manifest genuine effects. In contrast to Ellis et al. (2016)^56^ who identified an association between the IAT and self-report suicidal ideation, Rath et al. (2021)^57^ did not find such a correlation. It was important to note that both of these studies concentrated on patients in inpatient settings, and to the best of our knowledge, no earlier research has explored the link between entrapment and the IAT. One potential explanation for our findings is that the IAT may be more closely linked to behavior strongly related to suicide, such as self-harm^51^. In the field of suicide research, there is a need for objective measures owing to potential limitations in self-report methods^87^. However, it is worth noting that the results concerning the IAT and its connection to suicidality haven’t consistently been replicated, which prompts a discussion on the usefulness of the IAT for assessing suicide risk. Additional research and validation are necessary to better grasp the role of the IAT in suicide research and risk assessment.

The IMV-Model suggests that suicidal ideation develops from defeat, resulting in entrapment, resulting in acts. The current study did find some evidence for links between risk factors of the different stages, however, as our study was cross-sectional, results related to the IMV-Model should be interpreted with caution. The current study found support for relations between defeat, entrapment and suicidal ideation in revealing correlations between defeat and entrapment and in a logistic regression model entrapment predicted SI. The strong correlation (*r* = .82) between defeat and entrapment might indicate that, in a population of young healthy adults, defeat and entrapment are similarly linked to SI, and it is difficult to define suicidal risk ‘stages’, as proposed by O’Connor & Kirtley (2018)^13^. Importantly, the strong correlation between defeat and entrapment prohibited us from testing which psychological variables specifically moderate the defeat or entrapment and we therefore limited our analyses to the relation between entrapment and ideation/self-harm.

Our results further support the relation between depressive symptom severity and suicidality, which has been indicated in several previous papers^88,89^. Depressive symptom severity showed strong correlations between defeat/entrapment, suicidal ideation and self-harm in a sample of normal university students, and the regression model for self-harm including depression severity as a predictor revealed an effect of the death/suicidal IAT on self-harm, a relation that was not detected in the simple correlation analyses uncorrected for depression. Therefore, depression was shown to have a notable role in earlier stages, and its influence also affects later stages and related risk and protective factors of these stages of the suicidal process. Our results support the position of depression as a background factor of the *premotivational* phase in the IMV-Model.

Further, our moderation analyses indicated that self-compassion and the death/suicidal IAT are not moderators between entrapment and SI, and that mindfulness moderates this relation only on a trend level. Mindfulness has further shown a direct link to entrapment, especially external entrapment which might suggest mindfulness to be an individual protective factor linked to the motivational phase of the IMV-Model, and a moderator with a trend influence on the process from entrapment to suicidal ideation. The death/suicidal IAT as well as self-compassion were found to link to self-harm, and they are therefore associated with the volitional phase of the IMV-Model. In explorative analyses they were, however, not found to moderate the relation between entrapment and self-harm.

The current study did not reveal new moderators of escalating suicidal risk. Mindfulness could, however, be linked to the *motivational* phase of the IMV-Model, and self-compassion and d/s IAT to the *volitional* phase of the IMV. Concluding that mindfulness does indeed link to earlier phases of the suicidal process whereas self-compassion and the death/suicidal IAT link to later stages. Furthermore, depression severity should be considered as a component of the IMV-Model.

### Limitations

The current study has some limitations. First, although not surprisingly, a substantial portion of individuals with SI also engaged in self-harm, we therefore cannot make clear statements about determinants specific to SI or self-harm. Second, the current study is of cross-sectional nature and can therefore not give any indication about the predictive value of mindfulness, self-compassion and the death/suicidal IAT on future suicidality. Our static data cannot capture the dynamic temporal processes described in the IMV model. Therefore, future longitudinal research and ambulatory assessment are strongly needed to reveal valid determinants for the prediction of suicide and test the temporal hypotheses of the IMV model. Third, we did not measure mental illnesses including a current diagnosis, therefore we possibly compared individuals with a diagnosis of a mental disorder to individuals who without. However, we conducted and corrected for depression severity. Fourth, the measurement of self-harm did not allow us to separate NSSI and self-harm. Although NSSI is a risk factor for volitional phase in the IMV model, it does not reflect the behavioral enactment of this phase. Therefore, using a broad and mixed self-harm variable as a proxy of the volitional phase can be defective. This study provided a realistic representation of self-harm within a university population, where explicit suicide attempts were infrequent (6/94), however, future research should distinguish between NSSI and suicidal self-harm or attempts when examining the volitional phase of IMV model. The relatively modest sample size of the present study may limit the statistical power, especially for logistic regression and moderation analyses, which are statistically demanding and require larger samples to reach the stability of parameter estimates. The sensitivity power analyses to estimate the detectable effect sizes (see the supplementary Table) also suggested that the observed effects in the logistic regression and moderation models were smaller in magnitude than what they could detect. Therefore, this study is underpowered and has yet to reach a definitive conclusion regarding the small effects. Given the exploratory nature and the limited conclusiveness regarding small effects, these findings should be interpreted cautiously. Future research with larger and more balanced samples is necessary to replicate these findings.

## Conclusion

The current study identified self-compassion as a protective factor for self-harm and suicidal risk behaviour. In addition, mindfulness was identified as a factor associated with early cognitive and psychological states through a risk factor for suicide, namely entrapment. These constructions could therefore serve as promising targets for preventive intervention in the suicidal process. Weaker implicit associations with death/suicide were found in individuals with self-harm behaviour which might reflect an avoidance of death-related materials which may mirror the functionality of self-harm behaviour.

## Supplementary Information

Below is the link to the electronic supplementary material.

Supplementary Material 1

## Data Availability

The datasets of the current study are available upon reasonable request.

## References

[1] World Health Organization. *Mental Health of Adolescents* (2024). https://www.who.int/news-room/fact-sheets/detail/adolescent-mental-health

[2] World Health Organisation. *Suicide a leading cause of death among young adults in high-income countries* (2014). https://www.euro.who.int/en/health-topics/noncommunicable-diseases/mental-health/news/news/2014/09/suicide-a-leading-cause-of-death-among-young-adults-in-high-income-countries

[3] Mortier, P. et al. The prevalence of suicidal thoughts and behaviours among college students: a meta-analysis. *Psychol. Med.* **48**, 554–565. 10.1017/s0033291717002215 (2018). 28805169 10.1017/S0033291717002215

[4] Zhao, M. et al. Risk factors for suicidality among college students: A systematic review and meta-analysis. *J. Affect. Disord.* **382**, 567–578. 10.1016/j.jad.2025.04.137 (2025). https://doi.org/https://doi. 40280440 10.1016/j.jad.2025.04.137

[5] Mortier, P. et al. Suicidal thoughts and behaviors among First-Year college students: results from the WMH-ICS project. *J. Am. Acad. Child. Adolesc. Psychiatry*. **57**, 263–273e261. 10.1016/j.jaac.2018.01.018 (2018). 29588052 10.1016/j.jaac.2018.01.018 PMC6444360

[6] Borrill, J., Fox, P., Flynn, M. & Roger, D. Students who self-harm: coping style, rumination and alexithymia. *Counselling Psychol. Q.* **22**, 361–372 (2009).

[7] Toprak, S., Cetin, I., Guven, T., Can, G. & Demircan, C. Self-harm, suicidal ideation and suicide attempts among college students. *Psychiatry Res.* **187**, 140–144 (2011). 21040980 10.1016/j.psychres.2010.09.009

[8] Sivertsen, B. et al. Suicide attempts and non-suicidal self-harm among university students: prevalence study. *BJPsych Open.* **5**, e26. 10.1192/bjo.2019.4 (2019). 31068238 10.1192/bjo.2019.4 PMC6401540

[9] Chan, M. K. Y. et al. Predicting suicide following self-harm: systematic review of risk factors and risk scales. *Br. J. Psychiatry*. **209**, 277–283. 10.1192/bjp.bp.115.170050 (2016). 27340111 10.1192/bjp.bp.115.170050

[10] Kiekens, G. et al. The associations between non-suicidal self-injury and first onset suicidal thoughts and behaviors. *J. Affect. Disord.* **239**, 171–179. 10.1016/j.jad.2018.06.033 (2018). https://doi.org/https://doi. 30014957 10.1016/j.jad.2018.06.033

[11] O’Connor, R. C. The integrated motivational-volitional model of suicidal behavior. *Crisis* **32**, 295–298. 10.1027/0227-5910/a000120 (2011). 21945841 10.1027/0227-5910/a000120

[12] O’Connor, R. C. & Portzky, G. The relationship between entrapment and suicidal behavior through the lens of the integrated motivational–volitional model of suicidal behavior. *Curr. Opin. Psychol.* **22**, 12–17 (2018). 30122271 10.1016/j.copsyc.2017.07.021

[13] O’Connor, R. C. & Kirtley, O. J. The integrated motivational-volitional model of suicidal behaviour. *Philos. Trans. R Soc. Lond. B Biol. Sci.* **373**10.1098/rstb.2017.0268 (2018). 10.1098/rstb.2017.0268 PMC6053985 30012735

[14] Colmenero-Navarrete, L., García-Sancho, E. & Salguero, J. M. Relationship between emotion regulation and suicide ideation and attempt in adults and adolescents: A systematic review. *Arch. Suicide Res.* **26**, 1702–1735. 10.1080/13811118.2021.1999872 (2022). 34821201 10.1080/13811118.2021.1999872

[15] Dickhoff, J. et al. Relationship between social cognition, general cognition, and risk for suicide in individuals with a psychotic disorder. *Schizophr Res.* **231**, 227–236. 10.1016/j.schres.2021.02.024 (2021). 34000502 10.1016/j.schres.2021.02.024

[16] Ferrer, I. et al. I cannot read your eye expression: suicide attempters have difficulties in interpreting complex social emotions. *Front. Psychiatry*. **11**, 543889. 10.3389/fpsyt.2020.543889 (2020). 33240116 10.3389/fpsyt.2020.543889 PMC7683427

[17] Khurana, A. & Romer, D. Modeling the distinct pathways of influence of coping strategies on youth suicidal ideation: a National longitudinal study. *Prev. Sci.* **13**, 644–654. 10.1007/s11121-012-0292-3 (2012). 23054197 10.1007/s11121-012-0292-3

[18] Quintana-Orts, C., Mérida-López, S., Rey, L., Neto, F. & Extremera, N. Untangling the emotional Intelligence-Suicidal ideation connection: the role of cognitive emotion regulation strategies in adolescents. *J. Clin. Med.* **9**10.3390/jcm9103116 (2020). 10.3390/jcm9103116 PMC7599750 32993163

[19] Wang, K., Weiss, N. H., Pachankis, J. E. & Link, B. G. Emotional clarity as a buffer in the association between perceived mental illness stigma and suicide risk. *Stigma Health*. **1**, 252–262. 10.1037/sah0000032 (2016). 28090587 10.1037/sah0000032 PMC5223270

[20] Hoge, E. A. et al. Emotion-related constructs engaged by mindfulness-based interventions: A systematic review and meta-analysis. *Mindfulness (N Y)*. **12**, 1041–1062. 10.1007/s12671-020-01561-w (2021). 34149957 10.1007/s12671-020-01561-w PMC8210838

[21] Per, M., Simundic, A., Argento, A., Khoury, B. & Heath, N. Examining the relationship between Mindfulness, Self-Compassion, and emotion regulation in Self-Injury. *Arch. Suicide Res.* **26**, 1286–1301. 10.1080/13811118.2021.1885534 (2022). 33596395 10.1080/13811118.2021.1885534

[22] Eichholz, A. et al. Self-compassion and emotion regulation difficulties in obsessive-compulsive disorder. *Clin. Psychol. Psychother.* **27**, 630–639. 10.1002/cpp.2451 (2020). 32222000 10.1002/cpp.2451

[23] Inwood, E. & Ferrari, M. Mechanisms of change in the relationship between Self-Compassion, emotion Regulation, and mental health: A systematic review. *Appl. Psychol. Health Well Being*. **10**, 215–235. 10.1111/aphw.12127 (2018). 29673093 10.1111/aphw.12127

[24] Kabat-Zinn, J. *Wherever You go, There You Are: Mindfulness Meditation in Everyday Life* (Hyperion Books, 1994).

[25] Neff, K. & Dahm, K. in *Handbook of Mindfulness and Self-Regulation* 121–137 (2015).

[26] Deng, Y. Q. et al. The role of mindfulness and self-control in the relationship between mind-wandering and metacognition. *Pers. Indiv. Differ.* **141**, 51–56. 10.1016/j.paid.2018.12.020 (2019).

[27] Bluth, K. et al. Reducing suicide ideation in transgender adolescents with mindful Self-Compassion: an open trial. *Mindfulness* **15**, 3107–3128. 10.1007/s12671-024-02421-7 (2024).

[28] Rabasco, A., Wallace, G. T. & Andover, M. Mindfulness for reducing everyday suicidal thoughts (Mind-REST): A daily mindfulness intervention for adults with suicidal ideation. *Cogn. Therapy Res.* **49**, 155–168. 10.1007/s10608-024-10516-7 (2025). 10.1007/s10608-024-10516-7 PMC12392264 40894335

[29] Halladay, J. E. et al. Mindfulness for the mental health and Well-Being of Post-Secondary students: A systematic review and Meta-Analysis. *Mindfulness* **10**, 397–414 (2018).

[30] Parsons, D., Gardner, P., Parry, S. & Smart, S. Mindfulness-Based approaches for managing Stress, anxiety and depression for health students in tertiary education: a scoping review. *Mindfulness (N Y)*. **13**, 1–16. 10.1007/s12671-021-01740-3 (2022). 34539929 10.1007/s12671-021-01740-3 PMC8435111

[31] Lamis, D. A. & Dvorak, R. D. Mindfulness, nonattachment, and suicide rumination in college students: the mediating role of depressive symptoms. *Mindfulness* **5**, 487–496. 10.1007/s12671-013-0203-0 (2014).

[32] Collins, K. R. L., Stritzke, W. G. K., Page, A. C., Brown, J. D. & Wylde, T. J. Mind full of life: does mindfulness confer resilience to suicide by increasing zest for life? *J. Affect. Disord*. **226**, 100–107. 10.1016/j.jad.2017.09.043 (2018). 28968562 10.1016/j.jad.2017.09.043

[33] Argento, A., Simundic, A., Mettler, J., Mills, D. J. & Heath, N. L. Evaluating the effectiveness of a brief mindfulness activity in university students with Non-Suicidal Self-Injury engagement. *Arch. Suicide Res.* **26**, 871–885. 10.1080/13811118.2020.1841052 (2022). 33135590 10.1080/13811118.2020.1841052

[34] Schmelefske, E., Per, M., Khoury, B. & Heath, N. The effects of Mindfulness-Based interventions on suicide outcomes: A Meta-Analysis. *Arch. Suicide Res.* **26**, 447–464. 10.1080/13811118.2020.1833796 (2022). 33126844 10.1080/13811118.2020.1833796

[35] Petrovic, J., Bastien, L., Mettler, J. & Heath, N. L. The effectiveness of a mindfulness induction as a buffer against stress among university students with and without a history of Self-Injury. *Psychol. Rep.* **126**, 2280–2302. 10.1177/00332941221089282 (2023). 35473432 10.1177/00332941221089282 PMC10517589

[36] De Jaegere, E. et al. Mindfulness-Based cognitive therapy for individuals who are suicidal: A randomized controlled trial. *Arch. Suicide Res.* **28**, 1228–1248. 10.1080/13811118.2023.2282663 (2024). 37994872 10.1080/13811118.2023.2282663

[37] Galhardo, A., Cunha, M. & Pinto-Gouveia, J. Mindfulness-Based program for infertility: efficacy study. *Fertil. Steril.* **100**, 1059–1067. 10.1016/j.fertnstert.2013.05.036 (2013). 23809500 10.1016/j.fertnstert.2013.05.036

[38] Buitron, V., Hill, R. M. & Pettit, J. W. Mindfulness moderates the association between perceived burdensomeness and suicide ideation in adults with elevated depressive symptoms. *Suicide Life-Threatening Behav.* **47**, 580–588. 10.1111/sltb.12314 (2017). 10.1111/sltb.12314 27883204

[39] Shapira, L. B. & Mongrain, M. The benefits of self-compassion and optimism exercises for individuals vulnerable to depression. *J. Posit. Psychol.* **5**, 377–389 (2010).

[40] Sommers-Spijkerman, M., Trompetter, H., Schreurs, K. & Bohlmeijer, E. Pathways to improving mental health in Compassion-Focused therapy: Self-Reassurance, Self-Criticism and affect as mediators of change. *Front. Psychol.* **9**, 2442. 10.3389/fpsyg.2018.02442 (2018). 30568617 10.3389/fpsyg.2018.02442 PMC6290051

[41] Cleare, S., Gumley, A. & O’Connor, R. C. Self-compassion, self-forgiveness, suicidal ideation, and self-harm: A systematic review. *Clin. Psychol. Psychother.* **26**, 511–530. 10.1002/cpp.2372 (2019). 31046164 10.1002/cpp.2372

[42] Kelliher Rabon, J., Sirois, F. M. & Hirsch, J. K. Self-compassion and suicidal behavior in college students: serial indirect effects via depression and wellness behaviors. *J. Am. Coll. Health*. **66**, 114–122. 10.1080/07448481.2017.1382498 (2018). 28937937 10.1080/07448481.2017.1382498

[43] World Health Organisation. *Suicide* (2021). https://www.who.int/news-room/fact-sheets/detail/suicide

[44] Xavier, A., Pinto-Gouveia, J. & Cunha, M. The protective role of self-compassion on risk factors for non-suicidal self-injury in adolescence. *School Mental Health: Multidisciplinary Res. Pract. J.* **8**, 476–485. 10.1007/s12310-016-9197-9 (2016).

[45] Akin, A. & Akin, Ü. The predictive role of self-compassion on entrapment in Turkish university students. *Universitas Physiol.* **14**, 423–432 (2015).

[46] Cleare, S. *Exploring the Role of self-compassion in self-harm and Suicidal Ideation* (University of Glasgow, 2019).

[47] Nock, M. K. et al. Measuring the suicidal mind: implicit cognition predicts suicidal behavior. *Psychol. Sci.* **21**, 511–517. 10.1177/0956797610364762 (2010). 20424092 10.1177/0956797610364762 PMC5258199

[48] Harrison, D. P., Stritzke, W. G., Fay, N., Ellison, T. M. & Hudaib, A. R. Probing the implicit suicidal mind: does the Death/Suicide implicit association test reveal a desire to die, or a diminished desire to live? *Psychol. Assess.* **26**, 831–840. 10.1037/pas0000001 (2014). 24611787 10.1037/pas0000001

[49] Glenn, C. R., Millner, A. J., Esposito, E. C., Porter, A. C. & Nock, M. K. Implicit identification with death predicts suicidal thoughts and behaviors in adolescents. *J. Clin. Child. Adolesc. Psychol.* **48**, 263–272. 10.1080/15374416.2018.1528548 (2019). 30632815 10.1080/15374416.2018.1528548 PMC6405314

[50] Glenn, J. J. et al. Suicide and self-injury-related implicit cognition: A large-scale examination and replication. *J. Abnorm. Psychol.* **126**, 199 (2017). 27991808 10.1037/abn0000230 PMC5305619

[51] Toukhy, N. et al. Implicit or explicit self-associations with life and death? Predicting short-term self-injurious thoughts and behaviors among adolescents. *Death Stud.* **49**, 249–260. 10.1080/07481187.2024.2318601 (2025). 38393677 10.1080/07481187.2024.2318601

[52] Nock, M. K. & Banaji, M. R. Prediction of suicide ideation and attempts among adolescents using a brief performance-based test. *J. Consult Clin. Psychol.* **75**, 707–715. 10.1037/0022-006x.75.5.707 (2007). 17907852 10.1037/0022-006X.75.5.707 PMC2043087

[53] Wang, X. et al. Implicit measure of suicidal ideation in patients with depression. *Death Stud.* **46**, 1807–1813. 10.1080/07481187.2020.1850549 (2022). 33246393 10.1080/07481187.2020.1850549

[54] Millner, A. J. et al. Implicit cognitions as a behavioral marker of suicide attempts in adolescents. *Arch. Suicide Res.* **23**, 47–63. 10.1080/13811118.2017.1421488 (2019). 29482489 10.1080/13811118.2017.1421488

[55] Ruch, D. A. et al. Alterations in performance and discriminating power of the death/suicide implicit association test across the lifespan. *Psychiatry Res.* **335**, 115840. 10.1016/j.psychres.2024.115840 (2024). 38492262 10.1016/j.psychres.2024.115840 PMC12186299

[56] Ellis, T. E., Rufino, K. A. & Green, K. L. Implicit measure of Life/Death orientation predicts response of suicidal ideation to treatment in psychiatric inpatients. *Arch. Suicide Res.* **20**, 59–68. 10.1080/13811118.2015.1004483 (2016). 25923054 10.1080/13811118.2015.1004483

[57] Rath, D. et al. Predicting suicidal behavior by implicit associations with death? Examination of the death IAT in two inpatient samples of differing suicide risk. *Psychol. Assess.* **33**, 287–299. 10.1037/pas0000980 (2021). 33507799 10.1037/pas0000980

[58] Cull, J. G. & Gill, W. S. *Suicide Probability Scale* (1982).

[59] Cull, J. G. & Gill, W. S. Suicide probability scale. *Journal Consulting Clin. Psychology* (1988).

[60] Huisman, A., Smits, N. & Kerkhof, A. Signaleren Van suïcidaliteit Bij Jongeren Met de VOZZ-vragenlijst. *JGZ Tijdschrift Voor Jeugdgezondheidszorg*. **47**, 118–120 (2015).

[61] Gilbert, P. & Allan, S. The role of defeat and entrapment (arrested flight) in depression: an exploration of an evolutionary view. *Psychol. Med.* **28**, 585–598 (1998). 9626715 10.1017/s0033291798006710

[62] Baer, R. A., Smith, G. T., Hopkins, J., Krietemeyer, J. & Toney, L. Using self-report assessment methods to explore facets of mindfulness. *Assessment* **13**, 27–45 (2006). 16443717 10.1177/1073191105283504

[63] Bohlmeijer, E., Ten Klooster, P. M., Fledderus, M., Veehof, M. & Baer, R. Psychometric properties of the five facet mindfulness questionnaire in depressed adults and development of a short form. *Assessment* **18**, 308–320 (2011). 21586480 10.1177/1073191111408231

[64] Neff, K. D. The development and validation of a scale to measure Self-Compassion. *Self Identity*. **2**, 223–250. 10.1080/15298860309027 (2003).

[65] Neff, K. D. & Vonk, R. Self-compassion versus global self-esteem: two different ways of relating to oneself. *J. Pers.* **77**, 23–50. 10.1111/j.1467-6494.2008.00537.x (2009). 19076996 10.1111/j.1467-6494.2008.00537.x

[66] Greenwald, A. G., Nosek, B. A. & Banaji, M. R. Understanding and using the implicit association test: I. An improved scoring algorithm. *J. Personal. Soc. Psychol.* **85**, 197 (2003). 10.1037/0022-3514.85.2.197 12916565

[67] Cunningham, W. A., Preacher, K. J. & Banaji, M. R. Implicit attitude measures: Consistency, stability, and convergent validity. *Psychol. Sci.* **12**, 163–170 (2001). 11340927 10.1111/1467-9280.00328

[68] Lane, K. A., Banaji, M. R., Nosek, B. A. & Greenwald, A. G. Understanding and using the Implicit Association Test: IV: What we know (so far) about the method (2007).

[69] Moreno, M., Gutiérrez-Rojas, L. & Porras-Segovia, A. Implicit cognition tests for the assessment of suicide risk: A systematic review. *Curr. Psychiatry Rep.* **24**, 141–159 (2022). 35150387 10.1007/s11920-022-01316-5 PMC8852938

[70] Chiurliza, B. et al. Implicit measures of suicide risk in a military sample. *Assessment* **25**, 667–676 (2018). 27821459 10.1177/1073191116676363

[71] Beck, A. T. & Steer, R. A. *BHS, Beck Hopelessness Scale: Manual* (Psychological corporation, 1988).

[72] Rush, A. J. et al. The inventory for depressive symptomatology (IDS): preliminary findings. *Psychiatry Res.* **18**, 65–87 (1986). 3737788 10.1016/0165-1781(86)90060-0

[73] Baron-Cohen, S., Wheelwright, S., Hill, J., Raste, Y. & Plumb, I. The reading the Mind in the eyes test revised version: a study with normal adults, and adults with asperger syndrome or high-functioning autism. *J. Child. Psychol. Psychiatry*. **42**, 241–251 (2001). 11280420

[74] Wiebenga, J. X., Eikelenboom, M., Heering, H. D., van Oppen, P. & Penninx, B. W. Suicide ideation versus suicide attempt: examining overlapping and differential determinants in a large cohort of patients with depression and/or anxiety. *Australian New. Z. J. Psychiatry*. **55**, 167–179 (2021). 32847373 10.1177/0004867420951256

[75] Teismann, T. & Forkmann, T. Rumination, entrapment and suicide ideation: a mediational model. *Clin. Psychol. Psychother.* **24**, 226–234 (2017). 26663149 10.1002/cpp.1999

[76] Hair, J. F. Multivariate data analysis. (2009).

[77] Hayes, A. F. *Introduction to mediation, moderation, and conditional process analysis: A regression-based approach*. (The Guilford Press, 2013)

[78] Höller, I. et al. Defeat, entrapment, and suicidal ideation: Twelve-month trajectories. *Suicide Life-Threatening Behav.* **52**, 69–82. 10.1111/sltb.12777 (2022). 10.1111/sltb.12777 34142739

[79] Yusainy, C. & Lawrence, C. Relating mindfulness and self-control to harm to the self and to others. *Pers. Indiv. Differ.* **64**, 78–83. 10.1016/j.paid.2014.02.015 (2014). https://doi.org/https://doi.org/

[80] Heath, N. L., Carsley, D., De Riggi, M. E., Mills, D. & Mettler, J. The relationship between mindfulness, depressive symptoms, and non-suicidal self-injury amongst adolescents. *Archives Suicide Res.* **20**, 635–649 (2016). 10.1080/13811118.2016.1162243 26984524

[81] Collins, K. R., Best, I., Stritzke, W. G. & Page, A. C. Mindfulness and zest for life buffer the negative effects of experimentally-induced perceived burdensomeness and thwarted belongingness: implications for theories of suicide. *J. Abnorm. Psychol.* **125**, 704 (2016). 27175987 10.1037/abn0000167

[82] Chesin, M. S. & Jeglic, E. L. Factors associated with recurrent suicidal ideation among Racially and ethnically diverse college students with a history of suicide attempt: the role of mindfulness. *Archives Suicide Res.* **20**, 29–44 (2016). 10.1080/13811118.2015.1004488 26212484

[83] Luoma, J. B. & Villatte, J. L. Mindfulness in the treatment of suicidal individuals. *Cogn. Behav. Pract.* **19**, 265–276 (2012). 22745525 10.1016/j.cbpra.2010.12.003 PMC3383812

[84] Nock, M. K. Why do people hurt themselves? New insights into the nature and functions of Self-Injury. *Curr. Dir. Psychol. Sci.* **18**, 78–83. 10.1111/j.1467-8721.2009.01613.x (2009). 20161092 10.1111/j.1467-8721.2009.01613.x PMC2744421

[85] Chapman, A. L., Gratz, K. L. & Brown, M. Z. Solving the puzzle of deliberate self-harm: the experiential avoidance model. *Behav. Res. Ther.* **44**, 371–394 (2006). 16446150 10.1016/j.brat.2005.03.005

[86] Brereton, A. & McGlinchey, E. Self-harm, emotion regulation, and experiential avoidance: A systematic review. *Archives Suicide Res.* **24**, 1–24 (2020). 10.1080/13811118.2018.1563575 30636566

[87] Høyen, K. S. et al. Non-disclosure of suicidal ideation in psychiatric inpatients: rates and correlates. *Death Stud.* **46**, 1823–1831 (2022). 33586630 10.1080/07481187.2021.1879317

[88] Wiebenga, J. X. et al. Prevalence, course, and determinants of suicide ideation and attempts in patients with a depressive and/or anxiety disorder: A review of NESDA findings. *J. Affect. Disord.* **283**, 267–277 (2021). 33571797 10.1016/j.jad.2021.01.053

[89] Hawton, K., i Comabella, C. C., Haw, C. & Saunders, K. Risk factors for suicide in individuals with depression: a systematic review. *J. Affect. Disord.* **147**, 17–28 (2013). 23411024 10.1016/j.jad.2013.01.004

