# Supplementary material for: Psychological and cognitive-emotional moderators of suicidal ideation and self-harm in young adults
Source: Sci Rep. 2026 Jan 29;16:6625. doi: 10.1038/s41598-026-37127-4 (PMC12913718; doi:10.1038/s41598-026-37127-4)
Supplement: Supplementary file 1 — Supplementary Material 1 [file 41598_2026_37127_MOESM1_ESM.docx]

**Supplement Materials**

**METHOD – MEASURES**

**Death/suicide Implicit Association Test (d/s IAT)**

The death/suicide IAT started with an instruction about the task followed by five blocks. During each block words appeared either in the upper left or upper right corner of the screen, in the middle of the screen words (see Table 1) related to either one of the two categories in the corner appeared. Subjects had to indicate with pressing either “Q” for the left corner and “P” for the right corner. Words in the left/right corner were as follows: 1. Me/not me 2. Death/life 3. Me and death/not me and life 4. Life/death 5. Me and life/not me and death.

**Table S1**. Semantic stimuli of the d/s IAT.

| **Me (ik)** | **Not me (niet-ik)** | **Death (dood)** | **Life (leven)** |
| --- | --- | --- | --- |
| I (ik) | They (zij) | Die (sterven) | Alive (levend) |
| Myself (mezelf) | Them (hen) | Dead (dood) | Thrive (bloeien) |
| Self (zelf) | Their (hun) | Deceased (overleden) | Live (leven) |
| Mine (van mij) | Others (anderen) | Lifeless (levenloos) | Breathing (ademen) |
| My (mijn) | Theirs (van hen) | Suicide (zelfmoord) | Survive (overleven) |

Note. In the version used in the current study Dutch words were used. English words were translated using backwards translation, through a native Dutch and a bilingual Dutch-English speaker.

**Inventory of Depressive Symptomatology**

Self-Report (IDS-SR) The inventory of depressive symptomatology – self-report ^1^ measures current depressive severity. It contains 30 short items about depressive symptoms during the past seven days, each followed by four statements describing the severity of that symptom. A score between 0 and 3 was given for each item, 0 indicating no problems and 3 indicating severe problems. Twenty-seven out of the 30 items were scored (two are about either more or less appetite or weight gain or loss, where just one item was scored) which gave a sum score range between 0 and 81, with a higher score indicating more depressive symptoms. For the current study the total score (without the suicide item) was used.

**Beck Hopelessness Scale**

Previous studies showed a strong relation between hopelessness and suicidal ideation as well as self-harm^2-4^. We therefore included the Beck Hopelessness Scale, which is a well-known and often used self-report scale to measure severity of hopelessness ^5^. It contains 20 items with true-false rating scale (range: 0 – 20). The total score was calculated for the current study. The scale has shown good reliability (test-retest, r = 0.81) and internal consistency (alpha = 0.97 for depressive individuals and alpha = 0.79 for the control individuals ^6^.

**The Defeat Scale**

The Defeat Scale ^7^ was designed to depict a sense of failed struggle, and has often been used to measure the concept of defeat/humiliation, the feeling that “one has been brought down” ^8,9^. The scale consists of 16 items and assesses an individual’s perception of defeat in the previous seven days using a five-point Likert scale (ranging from 0 = “never” to 4 = “always”). The Defeat Scale shows high internal consistency with Alpha-coefficients of 0.94 in a student population ^7^.

**Reading the Mind in the Eyes Task (RMET)**

We administered the Reading the Mind in the Eyes Task (RMET^10^) as part of a pilot experiment for another large study. During the reading the mind in the eyes task, participants had to indicate the affective states, age and sex of 36 photographs depicting only the eye region of a person. All photographs were presented two times, in the first condition participants saw photographs accompanied by four emotional adjectives and in the second condition/control condition the photographs were accompanied by four adjectives indicating the age and sex (young man, young woman, old man, old woman) of a person. For each correct answer one point was assigned, the total correct scores (max. 32 per condition) were used in the current study. Facial affect recognition task was not related to any measure (*p*s>.15; see Table 2). Facial affect recognition task was not statistically significantly related to any measure in correlation analyses (*p*s>.15).

**RESULTS**

**Table S2**. Comparison of the scores on the suicide items between individuals with suicidal ideation and no suicidal ideation on the VOZZ and SPS scale.

|  |  | | **VOZZ** | |  | |  |
| --- | --- | --- | --- | --- | --- | --- | --- |
|  | **Item 7 (ever in lifetime SI)** | | **Item 9 (current SI)** | | **Item 10 (current SI)** | |  |
|  | noSI | SI | noSI | SI | noSI | SI | all |
| **SPS-SI** |  |  |  |  |  |  |  |
| noSI | 34 | 9 | 43 | 9 | 43 | 0 | 43 |
| SI | 15 | 36 | 44 | 6 | 42 | 8 | 50 |
| all | 44 | 44 | 87 | 6 | 85 | 8 |  |

Note. All participants whom indicated current suicidal ideation (in the previous seven days) on the VOZZ items also scored positive on suicidal ideation on the SPS scale. In total 36 subjects indicated to have suicidal ideation on both scales. And 34 indicated to have no ideation. 24 subjects indicated suicidal ideation only on one of the two scales we therefore did not include them in the analysis. Furthermore, we investigated the suicide-item of the IDS scale. Eight individuals indicated suicidal thoughts on the suicide-item of the IDS scale. All of them also indicated suicidal ideation on the SPS-SIS and on the VOZZ.

**Table S3**. Moderator analyses between entrapment and suicidal ideation with mindfulness, self-compassion the d/s IAT as moderators.

|  | **b¹** | **S.E.** | **z** | ***p*** | **95% Lower CI** | **95% Upper CI** |
| --- | --- | --- | --- | --- | --- | --- |
| *Mindfulness* | |  |  |  |  |  |
| Constant | 6.3743 | 4.3368 | 1.4698 | 0.1416 | -2.1256 | 14.8743 |
| Entrapment | -0.3241 | 0.2207 | -1.4687 | 0.1419 | -0.7566 | 0.1084 |
| Mindfulness² | -0.0619 | 0.0329 | -1.8797 | 0.0602 | -0.1264 | 0.0026 |
| **Entrapment x Mindfulness³** | **0.0042** | **0.0021** | **2.0339** | **0.042** | **0.0002** | **0.0082** |
| Depression | -0.0134 | 0.0644 | -0.2072 | 0.8358 | -0.1397 | 0.113 |
| *Self-compassion* | |  |  |  |  |  |
| Constant | 0.8774 | 2.586 | 0.3393 | 0.7344 | -4.1911 | 5.9459 |
| Entrapment | -0.1766 | 0.1831 | -0.9643 | 0.3349 | -0.5355 | 0.1823 |
| Self-compassion² | -0.6642 | 0.5654 | -1.1748 | 0.2401 | -1.7723 | 0.4439 |
| **Entrapment x Self-compassion³** | **0.1016** | **0.0559** | **1.816** | **0.0694** | **-0.0081** | **0.2112** |
| Depression | 0.0096 | 0.0639 | 0.15 | 0.8808 | -0.1156 | 0.1347 |
| *d/s IAT* |  |  |  |  |  |  |
| Constant | -1.5372 | 0.7268 | -2.115 | 0.0344 | -2.9617 | -0.1127 |
| Entrapment | 1.1364 | 0.613 | 1.8529 | 0.0639 | -0.0066 | 2.2339 |
| d/s IAT² | 1.0294 | 1.1382 | 0.9045 | 0.3658 | -1.2014 | 3.2603 |
| **Entrapment x d/s IAT³** | **-0.083** | **0.1025** | **-0.8093** | **0.4184** | **-0.2839** | **0.1179** |
| Depression | 0.0451 | 0.0705 | 0.6403 | 0.522 | -0.093 | 0.1833 |

Note. ^1^ Unstandardized beta coefficient.^2^ Moderator. ^3^ Interaction Effect.

**Table S4**. Spearman correlation analyses between internal, external entrapment and other variables

|  |  | Internal Entrapment | External Entrapment | Total Entrapment | Defeat | Hopeless  ness | Suicidal ideation (yes/no) | Self-harm (yes/no) | Self-entrapment | d/s IAT | Depression | Mindfulness |
| --- | --- | --- | --- | --- | --- | --- | --- | --- | --- | --- | --- | --- |
| Internal Entrapment | *r* | 1 | .868^**^ | 0.991^**^ | .853^**^ | .680^**^ | .697^**^ | .355^**^ | -.639^**^ | .210^*^ | .760^**^ | -.555^**^ |
|  | *p* |  | <0.001 | <0.001 | <0.001 | <0.001 | <0.001 | 0.001 | <0.001 | .044 | <0.001 | <0.001 |
|  | *N* | 94 | 94 | 94 | 91 | 94 | 70 | 92 | 94 | 92 | 93 | 94 |
| External Entrapment | *r* | .868^**^ | 1 | 0.932^**^ | .828^**^ | .678^**^ | .671^**^ | .347^**^ | -.664^**^ | .208^*^ | .770^**^ | -.609^**^ |
|  | *p* | <0.001 |  | <0.001 | <0.001 | <0.001 | <0.001 | 0.001 | <0.001 | .047 | <0.001 | <0.001 |
|  | *N* | 94 | 94 | 94 | 91 | 94 | 70 | 92 | 94 | 92 | 93 | 94 |

Note. ** indicates p<.05 **indicates p<.001.*

**Table S5.** Exploratory Moderation analyses between internal/external entrapment and suicidal ideation with mindfulness, self-compassion the d/s IAT as moderators.

| **Moderation Models** | ***b^1^*** | **S.E.** | **z** | ***p*** | **95% CI** | |
| --- | --- | --- | --- | --- | --- | --- |
|  |  |  |  |  | **Lower** | **Upper** |
| **Internal /External Entrapment (Moderator) 🡪 Suicidal ideation** | | | | | | |
| Constant | 6.2708 | 4.3156 | 1.453 | 0.1462 | -2.1877 | 14.7293 |
| Internal Entrapment | -0.5606 | 0.4086 | -1.3718 | 0.1701 | -1.3615 | 0.2403 |
| Mindfulness | -0.0635 | 0.0338 | -1.8792 | 0.0602 | -0.1298 | 0.0027 |
| **Internal Entrapment X Mindfulness** | **0.0072** | **0.0036** | **1.9911** | **0.0465** | **0.0001** | **0.0143** |
| Constant | 0.6259 | 2.461 | 0.2544 | 0.7992 | -4.1975 | 5.4493 |
| Internal Entrapment | -0.1566 | 0.312 | -0.5019 | 0.6157 | -0.7681 | 0.4549 |
| Self-entrapment | -0.6213 | 0.5729 | -1.0846 | 0.2781 | -1.7442 | 0.5015 |
| **Internal Entrapment X Self-entrapment** | **0.1345** | **0.0913** | **1.4732** | **0.1407** | **-0.0444** | **0.3134** |
| Constant | -1.4714 | 0.6304 | -2.334 | 0.0196 | -2.707 | -0.2358 |
| Internal Entrapment | 0.2726 | 0.085 | 3.2076 | 0.0013 | 0.106 | 0.4392 |
| d/s IAT | 0.9541 | 1.2829 | 0.7437 | 0.457 | -1.5603 | 3.4685 |
| **Internal Entrapment X d/s IAT** | **-0.1382** | **0.1916** | **-0.7213** | **0.4707** | **-0.5137** | **0.2373** |
| Constant | 5.8742 | 3.7021 | 1.5867 | 0.1126 | -1.3818 | 13.1301 |
| External Entrapment | -0.7484 | 0.445 | -1.6817 | 0.0926 | -1.6207 | 0.1238 |
| Mindfulness | -0.0551 | 0.0285 | -1.9327 | 0.0533 | -0.1111 | 0.0008 |
| **External Entrapment X Mindfulness** | **0.0092** | **0.0041** | **2.2745** | **0.0229** | **0.0013** | **0.0172** |
| Constant | 2.0957 | 1.9529 | 1.0731 | 0.2832 | -1.7319 | 5.9233 |
| External Entrapment | -0.5071 | 0.3825 | -1.3257 | 0.1849 | -1.2568 | 0.2426 |
| Self-entrapment | -0.8096 | 0.458 | -1.7677 | 0.0771 | -1.7072 | 0.088 |
| **External Entrapment X Self-entrapment** | **0.2518** | **0.1233** | **2.042** | **0.0412** | **0.0101** | **0.4934** |
| Constant | -0.9013 | 0.4953 | -1.8196 | 0.0688 | -1.8721 | 0.0695 |
| External Entrapment | 0.2998 | 0.088 | 3.4072 | 0.0007 | 0.1274 | 0.4723 |
| d/s IAT | 0.9415 | 0.9631 | 0.9776 | 0.3283 | -0.9461 | 2.829 |
| **External Entrapment X d/s IAT** | **-0.2421** | **0.2049** | **-1.1819** | **0.2373** | **-0.6436** | **0.1594** |
| **Internal /External Entrapment (Moderator) 🡪 Suicidal ideation *covariate depression*** | | | | | | |
| Constant | 6.4061 | 4.5467 | 1.409 | 0.1588 | -2.5052 | 15.3174 |
| Internal Entrapment | -0.5307 | 0.4129 | -1.2852 | 0.1987 | -1.34 | 0.2786 |
| Mindfulness | -0.0644 | 0.0349 | -1.8447 | 0.0651 | -0.1328 | 0.004 |
| **Internal Entrapment X Mindfulness** | **0.0069** | **0.0037** | **1.867** | **0.0619** | **-0.0003** | **0.0142** |
| Depression | -0.0033 | 0.0592 | -0.0558 | 0.9555 | -0.1194 | 0.1128 |
| Constant | 0.0358 | 2.6734 | 0.0134 | 0.9893 | -5.204 | 5.2756 |
| Internal Entrapment | -0.1753 | 0.3295 | -0.532 | 0.5948 | -0.8211 | 0.4705 |
| Self-entrapment | -0.5307 | 0.5877 | -0.9029 | 0.3666 | -1.6826 | 0.6212 |
| **Internal Entrapment X Self-entrapment** | **0.1304** | **0.0925** | **1.4089** | **0.1589** | **-0.051** | **0.3118** |
| Depression | 0.028 | 0.0617 | 0.4535 | 0.6502 | -0.0929 | 0.1489 |
| Constant | -1.7324 | 0.7512 | -2.3062 | 0.0211 | -3.2048 | -0.2601 |
| Internal Entrapment | 0.2171 | 0.1125 | 1.9299 | 0.0536 | -0.0034 | 0.4376 |
| d/s IAT | 0.9533 | 1.3024 | 0.7319 | 0.4642 | -1.5994 | 3.506 |
| **Internal Entrapment X d/s IAT** | **-0.1205** | **0.1981** | **-0.6081** | **0.5432** | **-0.5087** | **0.2678** |
| Depression | 0.0441 | 0.0674 | 0.6547 | 0.5127 | -0.0879 | 0.1761 |
| Constant | 5.6222 | 3.9541 | 1.4219 | 0.1551 | -2.1276 | 13.372 |
| External Entrapment | -0.7036 | 0.4532 | -1.5525 | 0.1205 | -1.5918 | 0.1847 |
| Mindfulness | -0.0548 | 0.0297 | -1.8482 | 0.0646 | -0.1129 | 0.0033 |
| **External Entrapment X Mindfulness** | **0.0085** | **0.0043** | **1.9911** | **0.0465** | **0.0001** | **0.0168** |
| Depression | 0.0223 | 0.0612 | 0.3644 | 0.7156 | -0.0976 | 0.1422 |
| Constant | 1.2961 | 2.4243 | 0.5346 | 0.5929 | -3.4554 | 6.0477 |
| External Entrapment | -0.4758 | 0.3832 | -1.2415 | 0.2144 | -1.2268 | 0.2753 |
| Self-entrapment | -0.6821 | 0.5058 | -1.3486 | 0.1775 | -1.6735 | 0.3092 |
| **External Entrapment X Self-entrapment** | **0.2293** | **0.1224** | **1.8733** | **0.061** | **-0.0106** | **0.4692** |
| Depression | 0.0286 | 0.0628 | 0.4552 | 0.6489 | -0.0945 | 0.1517 |
| Constant | -1.583 | 0.7314 | -2.1643 | 0.0304 | -3.0167 | -0.1494 |
| External Entrapment | 0.1746 | 0.1185 | 1.4731 | 0.1407 | -0.0577 | 0.4069 |
| d/s IAT | 0.99 | 0.9972 | 0.9927 | 0.3208 | -0.9645 | 2.9444 |
| **External Entrapment X d/s IAT** | **-0.2041** | **0.2072** | **-0.9854** | **0.3244** | **-0.6102** | **0.2019** |
| Depression | 0.0885 | 0.0684 | 1.2934 | 0.1959 | -0.0456 | 0.2226 |

Note. ^1^ Unstandardized beta coefficient.

**Figure S1**. Moderator analyses between self-harm and suicidal ideation and d/s IAT and self-compassion as moderators.


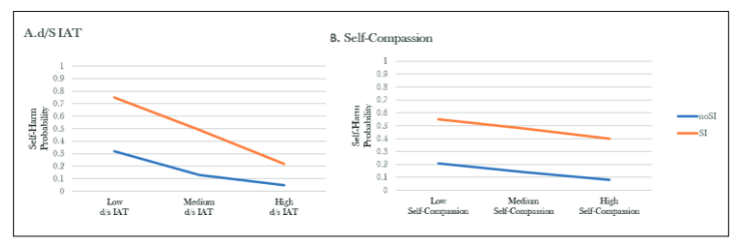


Note. As recommended by Hayes (2017) low medium and high scores of the independent and moderator variables indicate the 16th, 50th, and 84th percentiles. Probability of Self-Harm instead of log odds were indicated for to make interpretably more intuitive. Analyses are corrected for depression severity. SI=Suicidal Ideation, noSI=no Suicidal Ideation.

**Moderation analyses uncorrected for depression**

Results uncorrected for depression revealed similar results for mindfulness (*p* = .03, b = .004, z = 2.15), self-compassion (*p* = .05, b = .11, z = 1.94) and the d/s IAT (*p* = .36, b = -.09, z = -.92).

**Table S6.** Sensitivity power analyses

| **Hypothesis** | **N** | **Observed effect size** | **Detectable effect size (power=0.8)** |
| --- | --- | --- | --- |
| **Hypothesis 1:** SI ~ mindfullness+self-compassion+d/sIAT+depression | 70 | 0.95-1.32 (OR) | 2.19 |
| **Hypothesis 1:** SH ~ mindfullness+self-compassion+d/sIAT+depression | 92 | 0.05-1.10 (OR) | 2.67 |
| **Hypothesis 2:** Entrapment ~ mindfullness+self-compassion+d/sIAT+depression | 92 | 69.5% (R^2^) | 12% |
| **Hypothesis 3:** Moderation Entrapment X mindfulness | 70 | 0.243 (w) | 0.413 |
| **Hypothesis 3:** Moderation Entrapment X self-compassion | 70 | 0.217 (w) | 0.413 |
| **Hypothesis 3:** Moderation Entrapment X d/sIAT | 70 | 0.097 (w) | 0.413 |

Note: Sensitivity analysis of statistical power indicates that with an 80% statistical power set based on the current sample size, the minimum detectable effect size can be achieved for the overall model.

**Reference:**

1 Rush, A. J. *et al.* The inventory for depressive symptomatology (IDS): preliminary findings. *Psychiatry research* **18**, 65-87 (1986).

2 Lew, B. *et al.* Associations between depression, anxiety, stress, hopelessness, subjective well-being, coping styles and suicide in Chinese university students. *PLoS One* **14**, e0217372 (2019). <https://doi.org/10.1371/journal.pone.0217372>

3 Smith, J. M., Alloy, L. B. & Abramson, L. Y. Cognitive Vulnerability to Depression, Rumination, Hopelessness, and Suicidal Ideation: Multiple Pathways to Self-Injurious Thinking. *Suicide and Life-Threatening Behavior* **36**, 443-454 (2006). <https://doi.org/10.1521/suli.2006.36.4.443>

4 Steeg, S. *et al.* The exacerbating influence of hopelessness on other known risk factors for repeat self-harm and suicide. *J Affect Disord* **190**, 522-528 (2016). <https://doi.org/10.1016/j.jad.2015.09.050>

5 Beck, A. T., Weissman, A., Lester, D. & Trexler, L. The measurement of pessimism: the hopelessness scale. *Journal of consulting and clinical psychology* **42**, 861 (1974).

6 Bouvard, M., Charles, S., Guerin, J., Aimard, G. & Cottraux, J. Study of Beck's hopelessness scale. Validation and factor analysis. *L'encephale* **18**, 237-240 (1992).

7 Gilbert, P. & Allan, S. The role of defeat and entrapment (arrested flight) in depression: an exploration of an evolutionary view. *Psychological medicine* **28**, 585-598 (1998).

8 O'Connor, R. C. & Kirtley, O. J. The integrated motivational-volitional model of suicidal behaviour. *Philos Trans R Soc Lond B Biol Sci* **373** (2018). <https://doi.org/10.1098/rstb.2017.0268>

9 O'Connor, R. C. & Portzky, G. The relationship between entrapment and suicidal behavior through the lens of the integrated motivational–volitional model of suicidal behavior. *Current opinion in psychology* **22**, 12-17 (2018).

10 Baron-Cohen, S., Wheelwright, S., Hill, J., Raste, Y. & Plumb, I. The "Reading the Mind in the Eyes" Test revised version: a study with normal adults, and adults with Asperger syndrome or high-functioning autism. *J Child Psychol Psychiatry* **42**, 241-251 (2001).
